# Modified cantilever arrays improve sensitivity and reproducibility of nanomechanical sensing in living cells

**DOI:** 10.1038/s42003-018-0179-3

**Published:** 2018-10-24

**Authors:** Samadhan B. Patil, Rajai M. Al-Jehani, Hashem Etayash, Valerian Turbe, Keren Jiang, Joe Bailey, Walid Al-Akkad, Rania Soudy, Kamaljit Kaur, Rachel A. McKendry, Thomas Thundat, Joseph W. Ndieyira

**Affiliations:** 1 London Centre for Nanotechnology, 17-19 Gordon Street, WC1H 0AH and Division of Medicine, 5 University Street, WC1E 6JF, University College London, London, UK; 2 UCL Institute for Liver and Digestive Health, Royal Free Hospital and NHS Foundation Trust, London, NW3 2QG UK; 3 Department of Chemical and Materials Engineering, University of Alberta, Edmonton, AB T6G 2V4 Canada; 4 Faculty of Pharmacy and Pharmaceutical Sciences, University of Alberta, Edmonton, AB T6G 2E1 Canada; 5 Department of Medicine, Neuroscience and Mental Health Institute, University of Alberta, 530 Heritage Medical Research Centre, Edmonton, AB T6G 2S2 Canada; 6 Chapman University School of Pharmacy (CUSP), Harry and Diane Rinker Health Science Campus, Chapman University, Irvine, CA 92618-1908 USA; 7 Department of Chemistry, Jomo Kenyatta University of Agriculture and Technology, PO Box 62000, Nairobi, Kenya

## Abstract

Mechanical signaling involved in molecular interactions lies at the heart of materials science and biological systems, but the mechanisms involved are poorly understood. Here we use nanomechanical sensors and intact human cells to provide unique insights into the signaling pathways of connectivity networks, which deliver the ability to probe cells to produce biologically relevant, quantifiable and reproducible signals. We quantify the mechanical signals from malignant cancer cells, with 10 cells per ml in 1000-fold excess of non-neoplastic human epithelial cells. Moreover, we demonstrate that a direct link between cells and molecules creates a continuous connectivity which acts like a percolating network to propagate mechanical forces over both short and long length-scales. The findings provide mechanistic insights into how cancer cells interact with one another and with their microenvironments, enabling them to invade the surrounding tissues. Further, with this system it is possible to understand how cancer clusters are able to co-ordinate their migration through narrow blood capillaries.

## Abstract (web summary)

Samadhan Patil et al. report a new method for improving the sensitivity and reproducibility of mechanobiological measurements in malignant cancer cells. Their findings provide insight into the interaction of cells with each other and the microenvironment and may impact our understanding of metastasis.

## Introduction

There has been a growing appreciation in recent years that quantitative analysis of mechanical signals could be as useful as the chemical and electrical signaling generated from biochemical interactions. The remarkable ability of molecules to form complex structures and the mechanical forces^1–4^ arising from such interactions determine the collective mechanical response, thereby influencing a cascade of functional activities that include motility^5,6^, signaling, and homeostasis^7^. These mechanical forces play a vital role in embryonic development, as well as adult physiology^8,9^. In addition, there is mounting evidence that mechanical forces play an important role in disease states such as cancer as well as regulation of the immune response^8,10^.

Several techniques based on silicone rubber substrata^11^, micropatterned transparent elastomers^12^, and hydrogel cytometers^13^ have been specifically designed to quantify mechanical forces generated by biological systems. Despite their proven effectiveness, the sensitivity of these techniques is limited and fundamental gaps remain in our understanding of how molecules or cells collectively translate their interactions into mechanical forces. By virtue of their ability to resolve forces at the level of individual hydrogen bonds^14^_,_ mechanical sensors derived from micro-fabricated silicon cantilevers could potentially provide more sensitive strategies for quantifying the mechanical forces where both physiology and pathology come into play. These sensors are able to quantify interactions between ligands and capture molecules by tracking variations in resonant frequency due to mass loading^15–17^, adhesion forces^18^, and/or stress changes^19–22^. For example, cantilever technology has been used to unravel the mechanisms by which a near membrane surface layer regulates the molecular association kinetics for both mechanical force transduction and antimicrobial susceptibility^1^, solve a practical pharmacological problem of therapeutic monitoring in blood^23^, quantify protein interactions at femtomolar concentrations^24^, provide nanometrology of antibiotics^25^, and genotyping of cancer cells^26^. Moreover, this technology has demonstrated its ability as a nanoscopic toolbox allowing the visualization, in real-time, of pore-forming proteins^27^ and motor proteins^28^ as well as nanoscale characterization of plant cell walls^29^ and microbial cell surfaces^30,31^. The unique ability of nanomechanical sensors to measure forces at both the nano- and microscale level enables the mechanical properties of living cells to be simultaneously correlated with their biological activities such as, for example, when cells enter mitosis^32^ or bacteria form biofilms^33^. In spite of these advantages, cantilever technology suffers from a number of constraints, including reproducibility and reliability in signal response thus making its application in the medical field very challenging.

The label-free nanomechanical sensors have previously been investigated for their response to external forces arising from ligand attachments^3^; however, it remains unclear how the reproducibility of such signals depend on the physical location of chemically reacted regions. Here we describe a new approach to solve the problem of data reproducibility and reliability, which targets the signaling pathways. To produce biologically relevant, quantifiable, and reproducible signals, we took advantage of the bending moment in response to local stress caused by the recognition events between molecules or cells on the cantilever surface. We devised unique sets of capture molecule patching on the cantilever surface to unravel important aspects of how mechanical forces are relayed over both short and long length-scales. We hypothesized that signal reproducibility and sensitivity are determined by three factors. First, the hinge region (the anchoring area between the sensing element and pre-clamped solid support) is expected to be more sensitive to changes in stress than the free-end and so connectivity with the hinge region is likely to yield a large mechanical response. Second, the mechanical response is determined by continuous connectivity between the chemically transformed regions with each other and with the hinge region. This is regardless of whether all binding sites on the cantilever surface are occupied or not. Third, the signal sensitivity is determined by the chemistry and geometry of the sensing element so the design and structure of a nanomechanical sensor will determine the signal sensitivity. We validated these principles by using two powerful molecules; vancomycin (Van) as a model antibiotic compound and immunoglobulin G (IgG) as a naturally produced antibody both of which were dissolved in phosphate-buffered saline (PBS) solution. Van is currently in clinical use as one of the most powerful antibiotics in the battle against drug-resistant bacteria such as the “hospital superbug” methicillin-resistant *Staphylococcus aureus* as well as *Clostridium difficile* infections^34,35^. IgG is a major serum antibody responsible for the recognition, neutralization, or elimination of foreign invaders, including bacteria, fungi, and viruses. We also used intact human cells as models for the detection of larger entities and to understand the impact of mechanical connectivity in cell response.

We find that both healthy and cancer cells exert mechanical forces on the sensor, but the force exerted by the breast cancer cells is up to 200 times stronger than the normal healthy epithelial cells. Whereas cancer cells have identical receptors on their surface as the healthy cells, cancer cells are still highly effective even when they are outnumbered by 99 to 1. The high force that breast cancer cells exert on the sensor not only allows them to attach strongly to the surface but can also help them to penetrate through narrow blood capillaries—which gives us an insight into one of the ways that cancer is able to spread throughout the body. Until now it has not been clear how the network of cell-surface receptors and signaling pathways control the cell response, but our study suggests that the level of mechanical forces is location-specific and provides mechanistic insights into how cells interact with one another. This could further improve our understanding of how the interactions empower cancer cells to communicate with one another and with their microenvironments, enabling them to invade the surrounding tissues. The findings provide innovative insight into the specificity of cancer cell-derived mechanical response and will help us to introduce a new approach to pave the way for the development of more effective anticancer therapies to specifically target metastases. Further, this will help to better understand how cancer cells are able to co-ordinate migration to different parts of the body irrespective of the microenvironment.

## Results

### Pathways to force generation and propagation

**Fig. 1:**
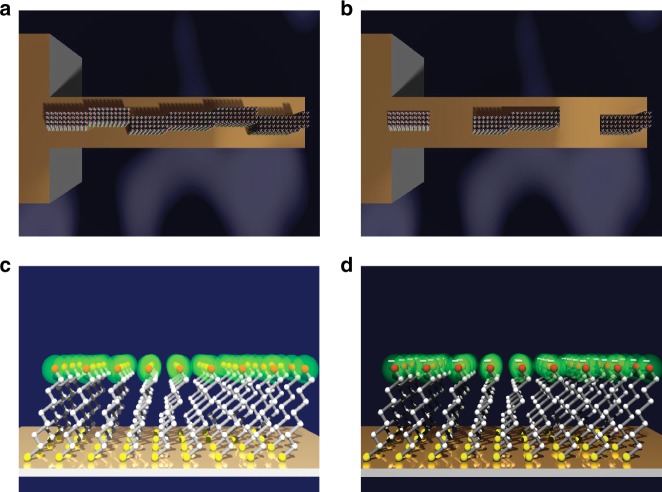
Evaluation of the impact of different receptor patterns on the generation of mechanical force. **a** Schematic diagram showing a random distribution with continuous connectivity between the capture molecules and the hinge region. **b** Random distribution of capture molecules with no continuity between each other and the hinge region. In **a** and **b**, the uncoated areas on the cantilevers (yellow orange) were passivated to block nonspecific interactions and the mechanical response was monitored in parallel using time-multiplexed optical beam detection on a single photodetector. **c** Schematic showing the principle of nanomechanical actuation on cantilevers coated with self-assembled monolayers (SAMs) of capture molecules with a rechargeable headgroup which is neutrally charged at pH 4.8 (green). **d** The corresponding SAMs, which are negatively charged at pH 9.0 (green with negative sign). In **c** and **d**, the total concentration of the ethanolic thiol solution of capture molecules was fixed at 2 mM. The results show that continuous mechanical connectivity is a relevant control parameter of signal sensitivity and reproducibility

**Fig. 2:**
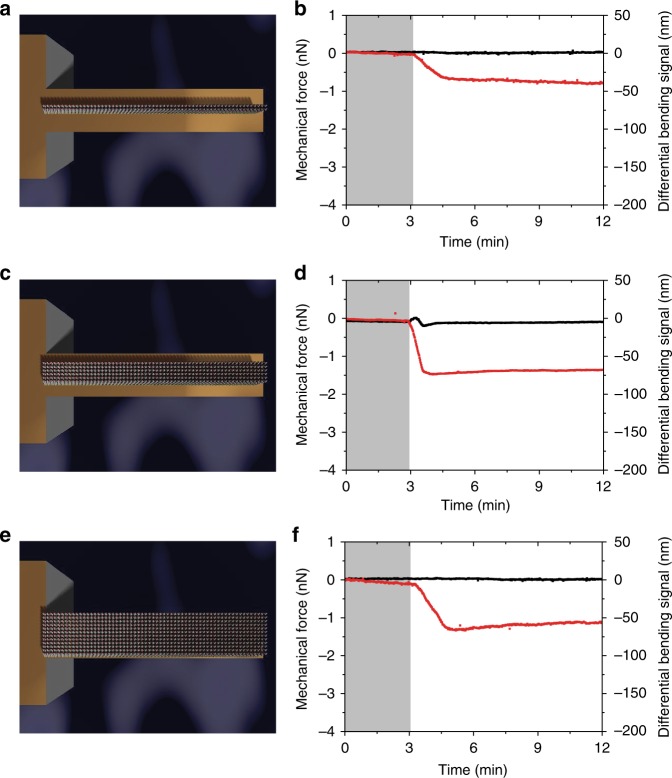
Geometrical widths of the regions covered by capture molecules, including the hinge region enhance force generation and propagation. **a**, **c**, **e** Schematic for the arrangement of sensing capture molecules (perpendicular sticks), whereas **b**, **d**, and **f** show the corresponding mechanical force generated (red) **a** where a narrow strip arrangement (30% of the total surface area) running centrally along the entire length of a nanomechanical cantilever sensor and continuous with the hinge. **c** Narrow strip arrangement (50% of the total surface area) running centrally along the entire length of a nanomechanical cantilever sensor and continuous with the hinge region. **e** A broad strip arrangement (100% of the total surface area) running centrally along the entire length of a nanomechanical cantilever sensor and continuous with the hinge region. In **b**, **d**, and **f**, the shaded areas represent the 3 min time frame during which the phosphate-buffered saline solution was injected without analytes to establish a baseline and the reference signal is shown in black. The negative signal corresponds to a compressive mechanical force on the nanomechanical cantilever sensor and the results demonstrate the impact of continuity of connectivity networks on the bio-signal processing. Further the results demonstrate that connectivity of capture molecules with each other and with the hinge region is key to the signal generation and propagation

**Fig. 3:**
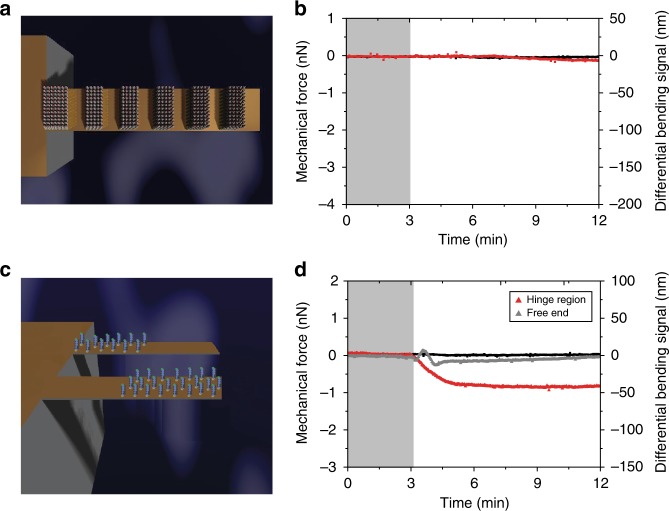
Networks of connectivity, which are discontinuous with each other and with the hinge region inhibit force generation and propagation. **a** The arrangement of capture molecules (perpendicular sticks) with strips transverse to the long axis of a cantilever sensor creating mechanical networks, which are discontinuous with each other and with the hinge region. **b** Shows the corresponding mechanical force generated (red). **c** Schematic representation of capture molecules (perpendicular sticks) arranged continuously from the hinge region and terminating just before the center (170 µm) of the cantilever sensor. Second, capture molecules arranged continuously from the free-end of the cantilever sensor and terminating just before the center (170 µm) of the cantilever sensor. **d** The corresponding mechanical force obtained when the capture molecules were arranged continuously from the hinge region and terminating just before the center (red) as well as arranged continuously from its free-end and terminating just before the center (gray). In **a** and **c**, the uncoated areas on the cantilevers (yellow orange) were passivated to block nonspecific interactions. In **b** and **d**, the shaded areas represent the 3 min time frame during which the phosphate-buffered saline solution was injected without analytes to establish a baseline. The negative signal is associated with compressive mechanical force on the gold top surface causing the cantilever to bend down and the reference signal is shown in black. The results demonstrate that connectivity of capture molecules with each other and with the hinge region is key to the signal generation and propagation

To explore the mechanisms underlying mechanical signaling, we first constructed different patterns of capture molecules on the cantilever surface (Fig. 1 and Supplementary Figs. 1–3). Figure 2a–f demonstrates how molecular machinery is positioned to transduce and propagate interactions between chemical entities into mechanical forces caused by electrostatic and/or steric interactions^36^. Here we find that an increase in the geometrical width of the regions covered by capture molecules, including the hinge region is characterized by a rise in response, confirming the assertion that force generation is a collective behavior of the ligand–receptor interactions. However, the crucial test is whether the connectivity between receptors and with the hinge region is necessary for the propagation of mechanical forces to be realized. To investigate this, we used cantilevers with identical surface coverage; differing only in the way the capture molecules are arranged. For example, in one instance the receptors were arranged in strips transverse to the long axis of the cantilever, creating a network, which is discontinuous with each other and with the hinge (Fig. 3a). Mechanical response from this arrangement was found to yield a small signal (Fig. 3b). In contrast, the arrays with receptors running centrally along the entire length of the cantilever and continuous with the hinge region gave rise to the largest signal response (Fig. 2).

**Fig. 4:**
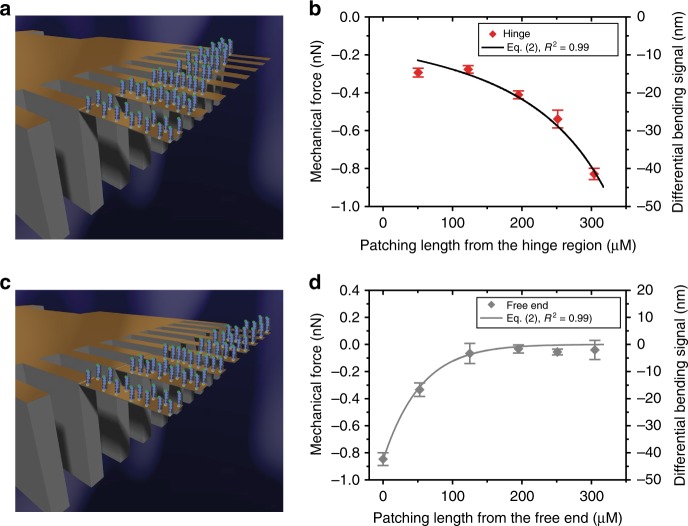
Mechanical force distribution as measured by mechanical sensors. **a** Capture molecules (perpendicular sticks) arranged continuously from the hinge region and terminating at varying distances from the free-end of a cantilever sensor. **b** The corresponding mechanical force obtained plotted as a function of the geometrical length of regions covered by the capture molecules shown by solid diamond symbols in red. **c** Capture molecules (perpendicular sticks) arranged continuously from the free-end of a cantilever sensor and terminating at varying distances from the hinge region. **d** The corresponding mechanical force obtained plotted as a function of the geometrical length of the capture molecules shown by solid diamond symbols in gray. In **a** and **c**, the plain areas (yellow orange) were passivated to block nonspecific interactions. In **b** and **d**, the solid lines connecting the diamond symbol data points were fitted to Eq. (2) to calculate the critical fraction of the region covered by capture molecules at which a signal is generated. The error bars shown represent the standard deviation obtained from four separate cantilever chips. The results demonstrate the impact of continuous connectivity network within capture molecules and with the hinge on the signal sensitivity and reproducibility

We next sought to determine whether capture molecules localized at the hinge region or free-end could improve the mechanical response or even identify a high-sensitive region for a targeted signal pathway to control the reproducibility of mechanical response. For these experiments, the geometrical width, and length of receptor patching was fixed at 100 µm and ≤170 µm, respectively, covering approximately 30% of the cantilever surface at either the hinge region or free-end (Fig. 3c). Figure 3d shows the outcome of the bending signals revealing a compressive force of −1000 ± 30 pN, which is much larger for the receptors disposed at the hinge region than at the free-end. This demonstrates that the geometrical effects have a large impact on the signal generation and, correspondingly, validate the conclusion that the hinge region is more sensitive to stress changes. Therefore, it should be possible to control the efficacy of the signals registered by the transducer by tuning the position of chemically reacted regions. Indeed, stress is determined by the geometric effects, which are responsible for the collective build-up of strain, therefore, we repeated the experiments in which the capture molecules’ coverage, *x* was systematically varied, where the plotted values are shown in the form of patching length (Fig. 4). The key aspect of this approach is to quantify the percolation threshold and to account for the large-scale mechanical consequences of stressed network formation. Figure 4a, b shows that by increasing the fraction of surface coverage of capture molecules at the hinge region from *x* = 0 to *x* = 0.1, a minimal mechanical signal is recorded, whereas from *x* = 0.1 to *x* = 1.0 there is an increase in response in direct proportion to the square of the geometric length covered by capture molecules in accordance with the power law relationship^37^. However, for the capture molecules starting at the free-end, increasing the surface coverage from *x* = 0 to *x* = 0.9 shows that a minimal signal response is measured, whereas from *x* = 0.9 to *x* = 1.0 the response is seen to increase exponentially (Fig. 4c, d). Remarkably, our results confirm that the geometrical distribution of capture molecules and the regions activated by local binding must be connected in terms of a network, including the hinge region to produce a mechanical signal. To quantify the correlation between signal pathways and propagation of mechanical forces, we used Eq. (1), whose detailed derivation is given in the Methods section.

1 $$\Delta F_{{\mathrm{eq}}} = F_{{\mathrm{max}}}\left( {\frac{{[{\mathrm{analyte}}]^n}}{{K_{\mathrm{d}}^n + [{\mathrm{analyte}}]^n}}} \right)\left( {\frac{{x - x_{\mathrm{c}}}}{{1 - x_{\mathrm{c}}}}} \right)^\alpha$$

Based on the percolation theory^38^, Eq. (1) holds true if *x* > *x*_c_. For a fixed analyte concentration, Eq. (1) reduces to Eq. (2) if *K*_d_ < [*analyte*] and so the net change in mechanical force is expressed as

2 $$\Delta F_{{\mathrm{eq}}} = F_{{\mathrm{max}}}\left( {\frac{{x - x_{\mathrm{c}}}}{{1 - x_{\mathrm{c}}}}} \right)^\alpha$$

To find the key parameters *x*_c_ and *α*, we carried out a least-squares fits of Eq. (2) with three fitting parameters (*F*_max_, *x*_c_ and *α*). Figure 4b shows the outcome, which reveals a percolation threshold *x*_c_ = 0.11 (~50 µm) and associated preference for a power *α* close to 1.8. The analysis shows that surface stress is indeed transduced collectively when a relatively large surface fraction is occupied. In particular, for *x* ≥ *x*_c_, the mechanical connectivity between chemically transformed regions is gradually established. Additionally, the short-range repulsive interactions, such as steric interactions between the nodes of the mechanical network, gives rise to signal transduction. In contrast, for *x* *<* *x*_c_ the network loses connectivity between chemically transformed regions and consequently leads to a breakdown in the collective behavior thereby inhibiting the generation and propagation of mechanical forces.

### Mechanical connectivity in cell response

**Fig. 5:**
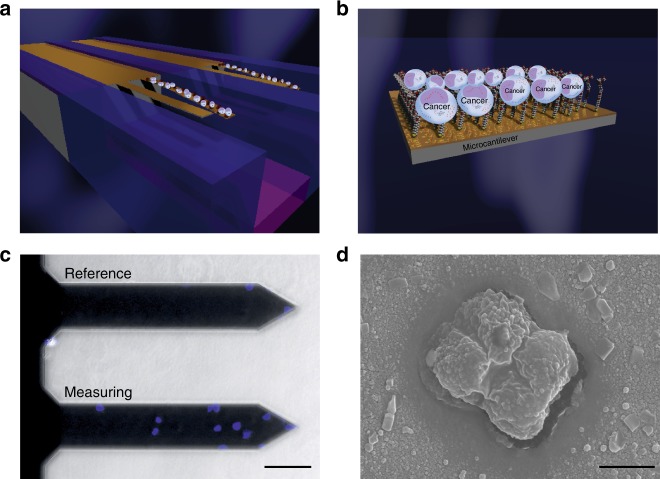
Nanomechanical detection of cancer cells in a model for breast cancer. **a** Schematic diagram showing attachment of malignant cells (white) to the cantilever surface. **b** The sketch shows a close-up image of the cell-receptor complex on the nanomechanical cantilever surface as used for the detection of mechanical response. **c** Merge image of DAPI-stained MDA-MB231 cells (blue) attached on a cantilever sensor, showing relatively fewer cells attached on an inert 6-mercapto 1-hexanol (MCH)-coated cantilever (reference) compared to a decapeptide-coated cantilever (measuring) obtained from malignant cancer cells suspended in PBS solution at 25 cells per ml. Scale bar, 100 μm. **d** Scanning electron microscopy image of a cancer cell attached to the measuring nanomechanical cantilever sensor. Scale bar, 1 μm. The results demonstrate that cantilever sensors have sufficient specificity and sensitivity to detect intact representative cancer cells at low concentrations without the need to use labels or sample processing

The network of chemical entities, such as cell-surface receptors and signal pathways, has long been known to control various aspects of cell behavior^10^. However, the principles and the mechanisms underlying mechanical connectivity between ligands and how it translates into a cellular response following activating interactions with neighboring cells remains unclear. To investigate this, we used the MDA-MB231 human breast carcinoma and MCF10 normal mammary epithelial cell lines. This is because these cells express different levels of receptors for the ligands such as decapeptide^39^; MDA-MB231 cells express a far greater number of receptors than normal healthy MCF10 cells^40,41^. To test if cancer cells bind selectively to ligands (Fig. 5a, b), decapeptide-coated cantilevers were exposed to the malignant cancer cells suspended in PBS solution at 25 cells per ml. Figure 5c, d shows the outcome where the merged image of 4′,6-diamidino-2-phenylindole (DAPI)-stained MDA-MB231 cells attached to the measuring cantilever reveals an average of 12 captured cells. In contrast, the control experiments showed an average of 2 cells, which indicates that the cantilevers have the sensitivity to successfully detect and quantify surface forces induced by specific cell–ligand interactions.

**Fig. 6:**
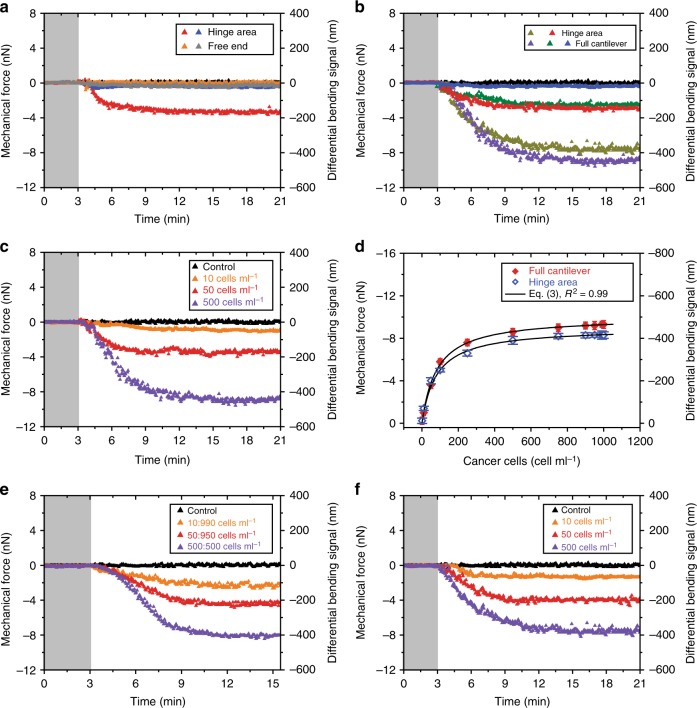
Mechanical connectivity in mechanobiology enhances cell response. **a** The mechanical force obtained from 25 cells per ml of cancer cells against ligands covering the hinge region only (red) and free-end (orange) of the cantilever. Corresponding mechanical force obtained from 25 cells per ml of epithelial cells against ligands covering the hinge region only (blue) and free-end (gray) of the cantilever. **b** The mechanical force obtained from 25 cells per ml of cancer cells against ligands covering the hinge region only (red) and fully covered cantilever (green). Corresponding mechanical force obtained from a higher cell concentration of 500 cells per ml covering the hinge region only (dark yellow) and a full cantilever (violet). Corresponding mechanical force obtained from 25 cells per ml of normal epithelial cells against ligands at a fully covered cantilever (blue). **c** The mechanical force at a fully covered cantilever obtained from 10 (orange), 50 (red), and 500 cells per ml (violet) of cancer cells. **d** A plot showing the measured mechanical force obtained from cancer cells against ligands covering the hinge region only (blue open diamond) or full cantilever (red solid diamond). The data are described by Eq. (3) for *K*_d_ = 80 ± 5 cells per ml (blue open diamond, covering the hinge region only) and for *K*_d_ = 81 ± 4 cells per ml (red solid diamond, fully covered cantilever). The error bars shown represent the standard deviation obtained from four separate cantilever chips each with eight cantilevers totaling 32 measurements. **e** The mechanical force against ligands at the hinge region obtained from a defined ratio of 1:99 (orange), 1:19 (red), and 1:1 (violet). **f** The mechanical force against ligands at hinge region obtained from 10 (orange), 50 (red), and 500 cells per ml (violet). In **a**–**c**, **e**, and **f**, the shaded areas represent the 3 min time frame during which the phosphate-buffered saline solution was injected without cells to establish a baseline. Negative signals are associated with compressive force and the reference signal is shown in black. The results provide mechanistic insights into the interactions of malignant cancer cells and may impact our understanding of metastasis

First, the mechanical signal generated by the bound cells was measured using cantilever arrays differing only in the position of ligands. Figure 6a shows the results upon injection of cancer cells initially fixed at 25 cells per ml. Analysis of the mechanical force exerted by cancer cells at the hinge region identified a compressive force of −3200 pN. Next, we performed the same experiment with ligands localized at the free-end of the cantilever. The measurements revealed a compressive force of −500 pN, which is approximately sixfold smaller than that resulting from the cells at the hinge region. These results are consistent with the findings obtained from the interaction of molecules on a cantilever surface, where a compressive force of −1000 pN was quantified for capture molecules disposed at the hinge region and approximately zero force measured for capture molecules positioned at the free-end (Fig. 3d). Given the cell concentration at both the hinge region and free-end is identical and that the interaction with the ligands is equally selective, regardless of their location on the cantilever surface, we can conclude that the relatively large signal in cell response is caused by the extrinsic sensitivity of the hinge region to stress. In addition, the approach demonstrates that mass effect alone is not sufficient to mediate considerable mechanical changes.

To test the specificity of cancer cell-derived compressive force at the hinge region, control measurements were performed using the non-neoplastic epithelial cells kept at a constant flow rate and concentration of 25 cells per ml to match the experimental conditions for the cancer cells. Normal epithelial cells gave rise to a small force of −500 pN regardless of whether the ligands were positioned at the hinge region or free-end of the cantilever (Fig. 6a). This reduction in the mechanical force is most likely due to the fact that these cells have far fewer receptors for the ligands on their surface^40,41^ compared to cancer cells, thus resulting in weakened cell–ligand interactions, confirming the specificity of the cell-derived compressive force at the hinge region. Further, the high mechanical forces exerted by cancer cells against the sensor could also help them to penetrate through narrow blood capillaries.

We next investigated whether the propagation of mechanical signaling is intimately related to the continuity of connectivity between the chemically transformed regions. For this we used cantilevers with ligands covering the hinge region only or the entire surface (full cantilever). The cancer cells were initially suspended in PBS at a dilution of 25 cells per ml. This particular concentration was chosen because (1) cells are capable of forming a percolating network at the cantilever surface and (2) the detection of cell response at ultralow concentration has important implications for the development of nanosensors with the requisite sensitivity and reproducibility necessary for clinical analysis of low levels of tumor cells in patients. Mechanical force of −4000 pN was measured with the ligands covering the hinge region only and a force of −3000 pN was measured when the entire cantilever was covered (Fig. 6b and Supplementary Fig. 4). The increased signal at the hinge region is consistent with a higher density of binding sites being occupied by cells, which are competing for a smaller number of binding sites, thus leading to enhanced connectivity between the reacted regions. This shows that although the total number of bound cells may be the same on the cantilever with either full or hinge only coverage, the hinge region is more efficient at amplifying signals from the reacted sites. Moreover, an additional experiment was performed using cancer cells at a higher concentration of 500 cells per ml where a signal of −7000 pN was observed for the hinge region and −9000 pN for the full cantilever coverage (Fig. 6b). Consequently, demonstrating that the forces generated on a cantilever surface are determined by the network of connectivity between activated regions regardless of whether all binding sites are occupied or not, in good agreement with the hypothesis.

To gain insight into the cell binding dynamics, we repeated the experiment using serial dilutions of cancer cells. With full cantilever coverage, the signal response was found to increase from −1000 to −9000 pN for the cell concentrations of 10 and 500 cells per ml, respectively (Fig. 6c). However, for concentrations between 500 and 1000 cells per ml the increase in signal response was less noticeable, varying from −9000 to −10 000 pN (Fig. 6d). To compare the variations in response on both types of cantilevers, we plotted the signals over the same concentration range of 10 to 1000 cells per ml (Fig. 6d). The mechanical force at the hinge region was substantially larger than the full cantilever when the cell concentration was <100 cells per ml. However, the mechanical response for a full cantilever was highest when the cell concentration was ≥100 cells per ml. Moreover, the response in both cases featured an initial steep rise in response followed by a plateau when most of the accessible surface-binding sites were occupied. The maximum force measured using only hinge coverage was −9000 ± 50 and −10 000 ± 80 pN for the full-coverage cantilevers. Moreover compared to −500 pN recorded for normal epithelial cells (Fig. 6a), the breast cancer cells exert a relatively much more force of up to 200 times stronger than the healthy cells. With such high force, it is possible to understand how cancer cells as a cluster are able to penetrate through narrow blood capillaries.

Although it is acknowledged that cancer cells may invade into the bloodstream during early stages of the primary tumor formation^42^, little is known about the impact of competing epithelial cells on their migration. Therefore, we next developed an approach to gain deeper insight into how normal healthy epithelial cells affect the ability of cancer cells to migrate through the fluid stream. This is a critical step needed for cancer cells in the blood to invade different parts of the body and form metastases. We directly quantitated mechanically the impact of the microenvironment on the migration of cancer by mixing breast cancer cells with non-neoplastic epithelial cells in a defined ratio of 1:99, 1:19, and 1:1. Upon exposure of these mixtures into a fluid stream, a compressive force of −2000, −4000, and −8000 pN was detected, respectively (Fig. 6e). As a measurement control, we repeated the experiments using cancer cells at concentrations of 10, 50, and 500 cells per ml but without competing epithelial cells. The data reveal a detection response of −1800, −4000, and −8000 pN (Fig. 6f). Inspection of the observed mechanical forces shows that the response in cancer cells is consistent irrespective of the microenvironment, an indication that cancer cells are able to recognize and distinguish other cancer cells with a similar structure while also ignoring normal healthy cells. This agreement demonstrates convincingly that the mechanics at the surface is strongly linked to the level of receptors expressed per cell and the strong interaction between cancer cells with membrane targets give rise to a direct link between cells, resulting in a continuous connectivity network just like an “interstate highway” to propagate forces over both short and long length-scales.

### Reliable and reproducible signal response with improved sensitivity

**Fig. 7:**
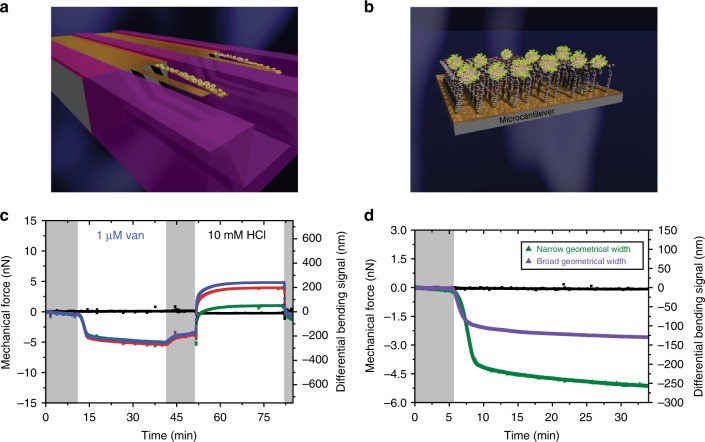
Mechanical connectivity and geometric width of cantilevers enhance force detection sensitivity. **a** Schematic diagram showing attachment of molecules (yellow) to the nanomechanical cantilever surface. **b** The sketch shows a close-up image of the ligand–receptor complex on the nanomechanical cantilever surface as used for the detection of mechanical response. In **a** and **b**, the uncoated areas on the cantilevers (yellow orange) were passivated to block nonspecific interactions and the mechanical response was monitored in parallel using time-multiplexed optical beam detection on a single photodetector. **c** The mechanical force from 1 µM Van against three separate experiments (green, red and blue). **d** The mechanical force from 1 µM Van against broad geometrical width (purple) and narrow geometrical width (green) of nanomechanical cantilever sensors. In **c** and **d**, the shaded areas represent the time frame during which the phosphate-buffered saline solution was injected without analytes to establish a baseline. The reference signal is shown in black and the negative signal is associated with compressive mechanical force on the gold top surface causing the cantilever to bend down. The results show that narrow cantilevers have the outstanding sensitivity and robustness to detect vancomycin. In addition, the results show that continuous mechanical connectivity is a relevant control parameter of signal sensitivity and reproducibility

Last, we attempted to determine whether altering the dimensions of the sensing element and surface chemistries, whilst maintaining the continuous connectivity with the hinge region, could improve the signal sensitivity and reproducibility. This is because (1) the threshold size of the cross-section area that is able to generate force decreases when the size of the sensing element is reduced; (2) impacting force on the sensor is more pronounced for a narrow sensing element and the narrower the sensing element, the less rigid it is and the more responsive it can be to the external forces; and (3) the hinge is more sensitive to the surface stress and therefore the signal response should increase as an inverse proportion to the size of the geometric width of the sensing element. Furthermore, the continuous connectivity network can be effected by the edging effects at the perimeter of the sensor itself and its impact would be more pronounced for narrow sensors. To demonstrate the feasibility that the sensor’s width could mediate more efficient continuous connectivity network and thus lead to enhanced signal sensitivity and reproducibility, we used 100 µm (broad geometrical width) and 70 µm (narrow geometrical width) against Van as a reporter molecule and Van-susceptible receptor (VSR) as its capture molecule (Fig. 7a, b). This particular system was chosen because it gives relatively large signals^23,24^ and so can be used to redefine the limit of recognition of biologically relevant systems.

**Fig. 8:**
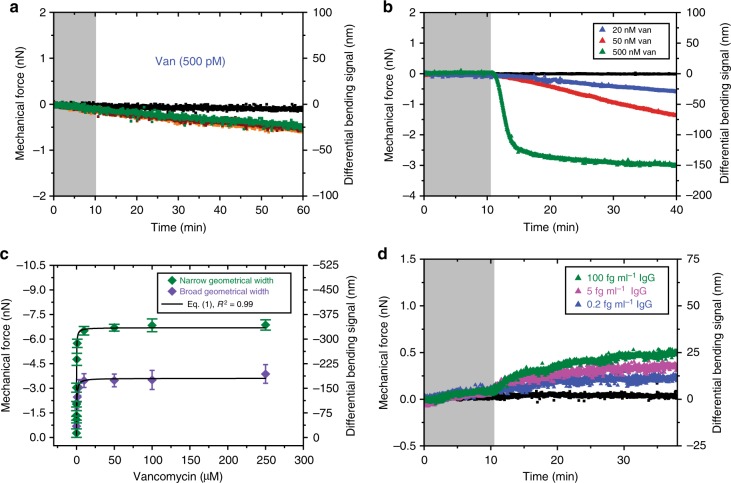
Combined sensor geometry and connectivity network elicit signal reproducibility and sensitivity. **a** The mechanical force obtained from 500 pM of Van. The orange, green, and wine colors represent data obtained from three separate nanomechanical cantilever sensors. **b** The mechanical force from 20 (blue), 50 (red), and 500 nM (green) Van. In **a** and **b**, the negative signal is associated with a compressive mechanical force on the gold top surface causing the cantilever to bend down. **c** The plot showing the measured mechanical force obtained from capture molecules using narrow geometrical width (green) and broad geometrical width (purple) of nanomechanical cantilever sensors. The data are described by Eq. (3) for *K*_d_ = 0.5 ± 0.2 µM (green diamond symbol, narrow nanomechanical cantilever sensors) and for *K*_d_ = 0.5 ± 0.2 µM (purple diamond symbol, broad sensing elements). The error bars shown represent the standard deviation of the mechanical force obtained from four separate cantilever chips each with eight cantilevers totaling 32 measurements. **d** The mechanical force obtained from 0.2 (blue), 5 (pink), and 100 fg per ml (green) immunoglobulin G (IgG). The positive signal is associated with tensile mechanical force on the sensing element causing the cantilever to bend upward. In **a**, **b**, and **d**, the shaded areas represent the time frame during which the phosphate-buffered saline solution was injected without analytes to establish a baseline. The reference signal is shown in black. The results show that by altering the dimensions of the sensing element and surface chemistries, whilst maintaining the continuous connectivity with the hinge, the limit of detection of proteins is significantly improved down to sub-femtogram levels without compromising the signal reproducibility or the need to use labels

To test whether the reproducibility of the mechanical signals in narrow cantilevers is activated by connectivity network between reacted regions and the hinge region, we performed measurements using 1 µM Van and three different narrow cantilevers fully coated with VSR capture molecules (Fig. 7c). The resulting response, whose magnitude is constant even when different cantilevers are used, demonstrates that the signal reproducibility originates from harnessing both the mechanical properties of the cantilever and connectivity network of chemically reacted regions. We next tested the effective performance of nanomechanical sensors by using two cantilevers comprising narrow and broad geometrical widths upon injection of 1 µM Van (Fig. 7d). The response featured by narrow cantilevers is −5000 pN, which is approximately three times larger than that from the broad cantilevers, where the net mechanical force is −2000 pN. The limit of detection, defined as the lowest concentration of Van, was determined by varying the concentration of Van in solution. Figure 8a shows the signal response obtained from three separate narrow cantilever sensors when they were exposed to 7 × 10^−10^ g per ml (500 pM) of Van in PBS solution at physiologically relevant pH 7.4. The observed mechanical force averaging −400 ± 30 pN with a signal-to-noise ratio (SNR) of 7 is by far beyond the experimental uncertainty. Moreover, the mechanical signals from the three separate sensors were highly consistent and almost identical over a 60 min time period. Similarly, we performed control experiments with broad cantilever sensors by following exactly the same protocol and observed no signal response at this ultralow Van concentration. Remarkably, the improved detection limits of Van down to 7 × 10^−10^ g per ml was found to be 4000 times better resolution than that obtained with the conventional methods such as Roche/Hitachi Cobas systems—currently in hospital use for the detection of antibiotics, which has reported the detection of 1.7 × 10^−6^ g per ml of Van^43^.

To further examine the impact of connectivity network on the performance of narrow cantilevers, we used different concentrations of Van (Fig. 8b). The results revealed that the continuous connectivity network correctly detected reactions reversibly at all concentrations and with successful label-free signal amplification. Moreover, the measurement at each antibiotic concentration was undertaken on at least four arrays (including broad and narrow cantilevers) and the resulting mechanical signals used to quantify the drug–target-binding interactions is summarized in Fig. 8c. To test whether the unexpectedly high and reproducible signals for Van could be replicated with other biologically relevant molecules, we performed additional measurements using IgG as the reporter molecule and anti-IgG antibody as its sensing receptor. Figure 8d shows the outcome of a tensile mechanical force of 200 pN with SNR of 5 and a limit of detection down to 2 × 10^−16^ g per ml (0.01 fM) of IgG. This level of sensitivity is significantly higher (10^4^-fold better) than that achieved with other widely used clinical assays for IgG such as the enzyme-linked immunosorbent assay, which has reported the detection limits of 1 × 10^−11^ g per ml (0.1 pM)^44^. The tensile mechanical force observed in Fig. 8d is probably due to van der Waals forces and hydrogen bonds, which might enable attractive interactions between bound complexes. To investigate further the mechanical response of narrow cantilever sensors, we chose different concentrations of IgG (0.2, 0.5, and 100 fg per ml) in PBS solution at pH 7.4 over the same time period. Consistent with Van, we found that the mechanical response scaled with the increasing IgG concentrations. As a reference, we repeated the experiments for the same concentrations of IgG by using broad cantilever sensors for which the measurements could not detect any mechanical response. At this point, it is possible that the enhanced force sensitivity of narrow cantilevers is driven by the network of signaling pathways and geometrical width of the sensing element.

### Quantifying the connectivity network on signal reproducibility

To gain further insight into the dynamics of connectivity with the hinge and propagation of mechanical forces, we made the assumption that the fraction of the surface coverage is unity. This enabled us to simplify expression (1) to obtain

3 $$\Delta F_{{\mathrm{eq}}} = F_{{\mathrm{max}}}\left( {\frac{{[{\mathrm{analyte}}]^n}}{{K_{\mathrm{d}}^n + [{\mathrm{analyte}}]^n}}} \right)$$

So to quantify the reliance of signal reproducibility on the mechanical connectivity with the hinge, the experimental data were modeled using Eq. (3) and the outcome of the fitted results revealed a striking similarity in *K*_d_ values of 0.5 ± 0.1 µM for broad and narrow cantilevers over a wide dynamic range of analyte concentrations. The maximum mechanical force generated by Van was −3500 ± 80 pN for the broad cantilevers and −6600 ± 20 pN for the narrow cantilevers (Fig. 8c). Moreover, the impact of continuity between ligands and the hinge area on the signal reproducibility was further quantified by using living cells (Fig. 6d). The calculated *K*_d_ of 80 ± 5 cells per ml for hinge region, representing a grafting area of only 30%, was found to be indistinguishable from a fully covered cantilever sensor (*K*_d_ = 81 ± 4 cells per ml).

To account for the consistent binding constants, we consider that whereas the limits of mechanical force sensitivity may be influenced by the size of the sensing element, however, the binding thermodynamics is not affected by the same consideration. This is because it is a geometry-independent quantity, which relies only on the interplay between mechanical forces and the concentration of analytes in the fluid stream. Consequently, the binding constant of an analyte is essentially identical whether it occurs in a three-dimensional (3D) solution or at two-dimensional (2D) surface. The difference in the binding constant, therefore, is as a result of the disparities in the mechanical forces and from any other effects that may alter the signal reproducibility. To lend support to this hypothesis, we compared the binding analysis for Van with conventional methods such as the 2D-surface plasmon resonance assays (*K*_d_ of 1.1 ± 0.4 μM^1^) and 3D-solution assays (*K*_d_ of 0.7 μM^45^), even though these techniques do not respond to the changes in force. The agreement of *K*_d_ values across a variety of techniques and between different geometrical widths of the sensing element is a further confirmation that the percolating network between capture molecules and with the hinge is the key determinant in establishing reproducible signals. To our knowledge, this represents the first use of nanomechanical sensors to quantitatively demonstrate that signal reproducibility is a specific function of connectivity network and lends support to the hypothesis that connectivity of capture molecules and/ or ligands with each other and with the hinge region is key to the signal generation and propagation.

## Discussion

The systematic experiments on model self-assembled monolayers (SAMs) and cancer cells suggest that the hinge and mechanical connectivity play a major role in mechanotransduction. We demonstrate that it is possible to decouple local and global effects on the signal response by using a rational design of connectivity networks at different length-scales. For the first time, we unravel a fundamental association between signaling and mechanical connectivity network, demonstrating that capture molecules arranged parallel to the long axis of a sensing element produce a larger response than those arranged in the transverse configuration. Our findings suggest that the forces generated are determined by the network of connectivity, which results from the short-range interactions between activated regions; regardless of whether all binding sites are occupied or not.

Moreover, we find that by altering the dimensions of the sensor and chemistries, the limit of detection of proteins is massively improved down to sub-femtogram levels without compromising the signal reproducibility or the need to use labels. This is confirmed by comparing an exactly solvable model to the experimental data where the outcome reveals a striking similarity in *K*_d_ values both for biomolecules and intact cells. This work will provide a blueprint for further studies to determine the role of the hinge and connectivity between activated regions to the reliability of mechanical response, which in turn will lead to the fabrication of ultrasensitive nanosensors for reproducible and sensitive assays of targets at ultralow concentrations. Perhaps, the most obvious application of the integration of percolating network in the analysis of various biological systems will lead to improved accuracy in diagnostic tests and insights into the role of mechanical changes in disease states, such as cancer, as well as in the regulation of the immune response.

More generally, we find that when ligands are optimally positioned on the cantilever surface, the detection of cancer cells is greatly improved down to 10 cells per ml. Moreover, a distinct response is generated between cancer cells and normal epithelial cells and that this can be accurately and reproducibly measured provided the activated regions are continuous with the hinge region and the global force network. The experiments show that the changes in forces triggered by cell–ligand interactions are determined by the specific location of the ligands on the cantilever surface. Interestingly, a negligible signal is obtained when ligands are positioned at the free-end of the cantilever, a region that is traditionally considered as a point load for maximum displacement. This means that mass effect alone is not sufficient to mediate considerable mechanical changes. We further demonstrate that the mechanical signals observed in mechanobiology on a microscale and biomolecular interactions on the nanoscale are consistent with the hypothesis that mechanotransduction operates via a universal phenomenon, which relies on a local short-range transduction mechanism. Consequently, our findings provide innovative insights into the physical aspects of cancer migration and open up novel strategies to prevent cancer from spreading. For example, if we can develop small particles that attach to the circulating cancer cells, these particles can both block the receptors and also lessen the interaction strength of cancer cells when they hit the surrounding tissues, this may take away the ability of cancer to spread and invade different parts of the body.

## Methods

### Design and synthesis of peptides

In this study, we used three capture molecules, VSR or l-Lys-d-Ala-d-Ala-OH found in bacteria cell envelopes, anti-mouse IgG antibody supplied by Sigma-Aldrich, UK and cancer cells targeting peptide (18-4). The peptide (18-4) is a short decapeptide engineered from the cancer homing peptide P160. The synthesis of VSR and decapeptide is briefly described. First, the decapeptides and VSR were coupled to additional cysteine residue HS(CH_2_CH)COOH and 11-mercaptoundecanoic acid (MUA) HS(CH_2_)_10_COOH, respectively. These alkanethiol moieties act as linkers between capture molecules and the gold-coated cantilever surface. The molecules were synthesized by solid phase methodology using standard Fmoc-protecting group chemistry. In the case of decapeptides, the process involved coupling the first amino acid to a 2-chlorotrityl resin (NovaBiochem, San Diego, CA) at fivefold excess using the *N*,*N* diisopropyl ethylamine at room temperature. The additional amino acids were added automatically using an automated peptide synthesizer (Tribute, Protein Technology, Inc., USA). The assembled decapeptide molecule was then released from the resin using a mixture of 90% trifluoroacetic acid, 9% dichloromethane, and 1% triisopropylsilane and washed with diethyl ether, dissolved in water and purified using reversed-phase high-performance liquid chromatography (RP-HPLC). The decapeptide molecules were isolated and selected by RP-HPLC retention time of synthetic peptides measured using Vydac C18 analytical column with a gradient by varying the mobile phase from 15 to 50% of acetonitrile in water (with 0.5% trifluoroacetic acid) where the mobile phase was allowed to flow at a rate of 1 ml min^−1^. For the VSR, the process involved using a commercially available pre-loaded Wang-d-Ala resins. The cleaved products were purified by RP-HPLC by varying the mobile phase from 5 to 95% of acetonitrile in water (with 0.5% trifluoroacetic acid). The assembled peptide products were characterized using nuclear magnetic resonance spectroscopy and high-resolution melting spectroscopy.

### Design and fabrication of cantilevers

IBM Rushlikon conventional nanomechanical cantilevers were supplied by Concentris GmBH. The narrow cantilevers were fabricated from a silicon wafer, defined by ultraviolet photolithography. The photoresist patterns were transferred to the device layer by reactive ion etching using standard microfabrication process of silicon on insulator (SOI), commonly employed in the production of semiconductor devices. The metal patterns were defined on top of the cantilever using lift-off and the silicon bulk etched with a deep-reactive ion etching. The cantilever arrays were released by removing the etch stop layer, resulting in fully free-standing cantilevers, accessible from both sides of the wafer. The cantilever arrays had the geometric width ranging between 70 and 100 µm whilst keeping the length and thickness at 500 and 1 µm, respectively. These dimensions were chosen because they allow a narrowed nanomechanical cantilever sensor to be used with the standard instrumentation of the detection system without the need for redesigning a new equipment. In addition, at this width, a narrow nanomechanical cantilever sensor is less likely to be affected by the capillary forces and static charges, which may render the requirements on the illumination alignment and the detector position more difficult. We therefore, examined the effect of reducing the geometric width of a typical cantilever sensor from 100 µm (broad geometrical width) to 70 µm (narrow geometrical width). For additional information, see the Supplementary Methods.

### Fabrication of micro-contact printing stamps

The silicon master mold was prepared using standard photolithography. Micro-contact printing (µCP) stamps made of poly(dimethylsiloxane) (PDMS) were prepared from the molds. The PDMS was chosen because, after polymerization and crosslinking, the solid PDMS is highly flexible and easy to peel off from the silicon or gold-coated surfaces. The PDMS polymer solution was freshly prepared by mixing silicone elastomer 184 base and silicone elastomer 184 curing agent (Dow Corning Corporation, USA) at a ratio 10:1 by weight in a disposable plastic container. The plastic container was then placed in a glass beaker inside a vacuum desiccator for 20 min to remove any gas bubbles. The resulting viscous solution was poured over the silicon master mold and baked at 75 °C in an oven for 1 h to enable crosslinking of the polymer and to transfer the etched pattern from the silicon master onto the solid PDMS. After cooling the imprinted solid PDMS stamps were peeled from the silicon master and trimmed to the required size.

### Cantilever sensor cleaning

Silicon cantilevers were cleaned by incubating in a freshly prepared piranha solution, consisting of H_2_SO_4_ and H_2_O_2_ (1:1) for 20 min. They were then briefly rinsed in ultrapure water followed by rinsing in pure ethanol before drying on a hotplate at 75 °C. The cantilevers were examined under an optical microscope to confirm their cleanliness and transferred to an electron beam evaporation chamber (BOC Edwards Auto 500, UK) where they were coated at a rate of 0.7 nm s^−1^ with a 2 nm layer of titanium, which act as an adhesion layer, followed by a 20 nm layer of gold. Once the required thickness of gold was obtained, the cantilever chips were left in the chamber for 1–2 h to cool under vacuum.

### Functionalization of cantilevers

*Printing of transduction arrays using µCP stamps*: To explore the mechanisms underlying mechanical signaling, we first constructed different patterns of capture molecules on the cantilever surface. Transduction arrays were printed on the gold top surface-coated cantilevers using SAMs of capture molecules as the printing ink. The protocol for printing the SAMs onto gold-coated cantilevers is summarized in Supplementary Figure 1. The PDMS stamp was first cleaned by rinsing in pure ethanol before it was dried under nitrogen gas. The stamp was then impregnated with SAMs by incubating it in a freshly prepared solution of SAMs of capture molecules in ethanol at a total concentration of 2 mM for 1 min. Excess SAMs of molecules were removed from the PDMS stamp by blowing nitrogen gas over the stamp. The impregnated stamp was then placed in a conformal contact with gold-coated surface of the cantilever for 2 min where a gentle pressure (using a one penny coin) was applied on the PDMS stamp to allow close contact with the cantilever surface so that the SAMs of capture molecules could diffuse from the PDMS stamp onto the cantilever surface to enable uniform molecular printing. The printing of cantilevers was carried out in an upside down configuration in which the PDMS stamp faced upwards, whereas the gold-coated cantilever surface faced downwards. The cantilever, which was initially oriented with an angle of tilt away from the surface horizontal, was carefully moved downwards until it was in full contact with the surface of the PDMS stamp. The PDMS stamp was removed after 2 min and the un-patterned areas on the cantilever surface were passivated for 20 min by inert SAM molecules known to resist biomolecule adsorption on surfaces. We initially used highly packed SAMs of MUA and mercaptohexadecanoic acid, where the geometrical length of the regions covered with capture molecules is fixed at 500 µm long. These SAMs were chosen due to their versatility in terms of the diversity of capture molecules that they can be attached to and for their ability to change surface charge under different pH conditions^36^. They, therefore represent ideal tools for investigating the propagation of mechanical forces caused by electrostatic and/ or steric interactions.

*Printing of arrays using dip-pen nanolithography*: The printing process involved using a sharp scanning atomic force microscopy (AFM) cantilever tip to transfer SAMs of capture molecules as the printing ink directly onto the designated cantilever surface and to create the desired pattern. The success of this approach was first tested on the gold-coated silicon substrates (Supplementary Figure 3d) before applying the same procedure to create transduction arrays on the nanomechanical cantilevers. An AFM cantilever tip functionalized with SAMs of capture molecules fixed at a total concentration of 2 mM was brought into contact with the gold-coated cantilever and slowly traced at a resolution of 256 lines per µm^2^ and at a frequency of 1 Hz. The low scan speed was necessary to enable precise delivery of SAMs to the surface via formation of a liquid meniscus. The printed pattern was then imaged using the same AFM cantilever tip using a scan speed of 10 Hz. The high scan speed was essential to prevent deposition of any additional SAMs during imaging. The un-patterned areas on the cantilever surface were passivated for 20 min by inert SAMs molecules known to resist biomolecule adsorption on surfaces. For additional information, see Supplementary Methods.

*Printing of transduction arrays using microcapillaries*: First a mixture of ethanolic thiol solutions was made using mercaptoundecyl tri(ethylene glycol) thiol (PEG) or (HS-C11-(Eg)_3_-OMe) and NHS (HS-C11-(Eg)_3_-OCH_2_-COONHS) (where Eg is an ethylene glycol group, Me is a methyl group, and NHS is the *N*-hydroxysuccinimide group) (ProChimia Surfaces, Poland). PEG and NHS were mixed using a defined ratio of 1:9 while keeping the total thiol concentration fixed at 1 µM in pure ethanol. The cantilevers were incubated in an array of eight glass microcapillaries filled in a random order with 1 µM ethanolic solutions of PEG/NHS mixture or pure PEG for 20 min, and then rinsed in pure ethanol and dried under nitrogen gas for 2 min. The PEG/NHS-coated nanomechanical cantilever sensor arrays were passivated by incubating in 2-[methoxypoly(ethyleneoxy) propyl]trimethoxysilane for 30 min followed by a rinse in pure ethanol. They were activated by using sodium acetate buffer (5 mM, pH 5.4) for 5 min at room temperature and placed inside a chamber where they were submerged in a droplet containing 100 µg per ml of anti-mouse IgG antibody (Sigma-Aldrich, UK) dissolved in PBS solution. The chamber was kept at 4 °C overnight to enable complete conjugation of NHS and IgG. To cap any unconjugated NHS molecules, the nanomechanical cantilevers were incubated in 1 M ethanolamine, pH 8.5, for 5 min at room temperature. This was followed by a wash in PBS solution. To coat the hinge area with the cancer-targeting peptides, the free-end of each cantilever was first coated with 1 mg per ml of SAMs of inert 6-mercapto 1-hexanol (MCH) solution in pure ethanol. This step process is necessary to prevent nonspecific adsorption of cells on the gold-coated surface. The cantilever chip was rinsed with PBS solution at pH 7.4 before drying in air. The uncoated hinge area on the cantilever surface was coated with cancer-targeting peptides by incubating the entire cantilever chip in 1 mg per ml of SAMs of decapeptide for 20 min followed by a rinse in PBS solution. In parallel, the reference cantilevers were coated with SAMs of MCH at 1 mg per ml. Subsequently, the cantilevers were passivated using PEGsilane to block nonspecific interactions at the silicon underside surface. This protocol was applied in the case of the free-end of the cantilever or full cantilever coverage. For the capture molecules of VSR, the process involved incubating individual cantilevers in an array of eight glass microcapillaries filled in a random order with 1 µM ethanolic solutions of SAMs of VSR and PEG for 20 min. This was followed by rinsing in pure ethanol and deionized water but without underside passivation.

### Cell culture and measurement

Two different cell lines were used in this study, the human breast cancer cell line MDA-MB231 and the human mammary epithelial cell line MCF10A. These cell lines were obtained from the American Type Culture Collection (ATCC) cell bank and were authenticated via optical microscope to check for their morphology, state, and fungal contamination (ATCC, Manassas, VA). In addition, the cell lines were tested for mycoplasma contaminations. MDA-MB231 cell line was grown in Dulbecco’s modified Eagle medium, supplemented with 10% fetal bovine serum, 1% glutamine, and 100 IU per ml penicillin-streptomycin, and MCF10A cell line was cultured in a minimal essential growth medium (Lonza, Cedarlane) supplemented with the same additives, where both cell lines were incubated at 37 °C in a 5% CO_2_–95% O_2_ incubator, and the growth media were exchanged every 48 h. To prepare the cells for mechanobiology, 100% confluent cells were detached from the culture flasks using manual cell scrapers without the use of trypsin to preserve cell structure and membrane proteins. The cell suspension was centrifuged, the culture medium removed, and the cells resuspended in PBS solution at pH 7.4. Subsequently, the cells were counted using a hemocytometer and then diluted to a stock concentration of 1000 cells per ml in PBS solution. Serial dilutions at working concentrations of 990, 950, 900, 750, 500, 250, 100, 50, 25, and 10 cells per ml were prepared from this stock solution. The attachment of the cells on the cantilever was facilitated by functionalizing the surface with cell homing decapeptides at a fixed concentration of 1 mg per ml. To investigate external forces arising from cell–ligand interactions, the cells suspended in PBS solution were injected into the cantilever chamber at a constant flow rate of 1 ml h^−1^.

### Fixation/imaging

Prior to fluorescence microscopy, cancer cells were fixed onto the cantilever surface after 10 min using 4% paraformaldehyde for 15 min before rinsing in PBS solution and the cell nuclei were stained with DAPI (blue) before imaging. Confocal micrograph stacks were acquired with a Zeiss Axioskop 40 and the images were captured with an Axiocam Zeiss Axiovision (version 4.8.2).

### Scanning electron microscopy

The stained cells on the cantilever were again rinsed in absolute ethanol (99.8%, Fisher Scientific UK Limited), after which the sample was dried in air. A five-nanometer gold coating was applied to the cantilever with the attached cells before scanning electron microscopy imaging. The imaging was performed using a Carl Zeiss XB1540 “Cross-Beam” focussed-ion-beam microscope.

### Quantifying the connectivity network on mechanical signals

To develop greater insight into the precise link between signaling pathways and propagation of mechanical forces, we made the assumption that the net mechanical response is a product of the local stress and connectivity between the capture molecules with each other and with the hinge region. These two separate and distinct effects as shown in Fig. 4b can be summarized using Eq. (1)^25^, which is based on the percolation theory^38^. Figure 4b shows values plotted in the form of patching length (500× µm, where 500 µm is the effective length of the cantilever). The percolation theory describes the build-up of stress following the connectivity of chemically transformed networks and the interactions between nodes of the network.

1 $$\Delta F_{{\mathrm{eq}}} = F_{{\mathrm{max}}}\left( {\frac{{[{\mathrm{analyte}}]^n}}{{K_{\mathrm{d}}^n + [{\mathrm{analyte}}]^n}}} \right)\left( {\frac{{x - x_{\mathrm{c}}}}{{1 - x_{\mathrm{c}}}}} \right)^\alpha$$

The first term is the Langmuir adsorption isotherm, which describes the strength of analyte–receptor complex interactions and the second term is the power law form describing the large-scale mechanical consequences of stressed network formation. *F*_eq_ is the equilibrium mechanical force, *F*_max_ is the maximum mechanical force when all accessible binding sites are fully occupied, *K*_d_ is the thermodynamic equilibrium dissociation constant, *n* is the stoichiometric coefficient of the reaction, and *K*_d_ is raised to the power *n* to ensure that the dimension of the concentration remains constant as *n* varies, *x* is defined as the measure of the regions covered by capture molecules on the cantilever expressed as a fraction of fully covered cantilever, *x*_c_ is the critical fraction of the region covered by capture molecules at which a signal is generated, and *α* is the power law associated with the collective behavior of chemically reacted regions required to generate a signal. Equation (1) whose validity has previously been verified^25^ holds true if *x* > *x*_c_. For a fixed concentration of the analyte, Eq. (1) is further simplified to obtain Eq. (2).

2 $$\Delta F_{{\mathrm{eq}}} = F_{{\mathrm{max}}}\left( {\frac{{x - x_{\mathrm{c}}}}{{1 - x_{\mathrm{c}}}}} \right)^\alpha$$

The hypothesis that the reproducibility in mechanical response is governed to a large extent by the continuity of connectivity between sensing ligands and the hinge, is so far purely phenomenological. To gain quantitative insight into the dynamics of connectivity with the hinge and propagation of mechanical forces, we made the assumption that the fraction of the surface coverage is unity. This enabled us to simplify expression (1) to obtain Eq. (3) expressed as

3 $$\Delta F_{{\mathrm{eq}}} = F_{{\mathrm{max}}}\left( {\frac{{[{\mathrm{analyte}}]^n}}{{K_{\mathrm{d}}^n + [{\mathrm{analyte}}]^n}}} \right)$$

Equation (3) offers a particular understanding of mechanical forces obtained from different dimensions of the sensing elements and the impact of mechanical connectivity with the hinge, which may help to design better analysis for the direct mechanical assays at ultralow concentrations.

### Measurement of force exerted by cells and biomolecules

The cantilever chip modified with decapeptide ligand and/or VSR was mounted in a sealed liquid chamber with a volume of ∼80 μl and placed in a temperature-controlled cabinet. The alignment of each light source onto the free-end of each cantilever was confirmed by heating the liquid chamber to 1 °C rise. All eight gold-coated cantilever arrays were found to undergo compressive downward bending because of the bimetallic effect caused by the differences in the expansion rates of silicon and gold. Care was taken to ensure that the optical alignment error was minimized to <5% between the minimum and maximum bending signals within the cantilever arrays. The mechanical force generated upon binding of cancer cells, Van or IgG on each of the eight cantilevers was measured in parallel and under identical environment using a Scentris (Veeco Instruments) optical beam device. The measurement protocol involved the following steps, (1) injecting PBS solution for 3 or 10 min to establish a baseline; (2) injection of cancer cells, Van, or IgG in PBS solution for 30–60 min; (3) PBS solution wash for 10–30 min to dissociate the bound complex; (4) a further washing step using 10 mM HCl/or 10 mM glycine-HCl, pH 2.5, for another 5–30 min to regenerate the surface; and (5) finally another PBS solution step for 5–10 min to restore the baseline signal. The injections of different concentrations of Van or IgG was achieved by using an automated pumping system (Model Genie Plus, Kent Scientific) at an optimized flow rate of 10–150 μl min^−1^. A constant flow rate of 1 ml h^−1^ was used in the case of cancer cells suspended in PBS solution. Artifacts that produce nonspecific signals were overcome by performing differential measurements. The raw data from four separate cantilever chips totaling 32 measurements in each experiment was analyzed to calculate the absolute bending deflections, *z*_abs_ (in nm). The differential equilibrium bending deflections (Δ*z*) was calculated by subtracting the in situ reference Δ*z*_ref_ (MCH or PEG) coated cantilever signals from the absolute mechanical response Δ*z*_mea_ (VSR, anti-IgG, and cancer-targeting-peptide-coated cantilevers). This was subsequently converted into differential equilibrium mechanical force *F* using the expression

4 $$F = k\Delta z$$

where *k* is the cantilever’s nominal spring constant. The nominal spring constant for broad IBM fabricated silicon cantilevers (1 µm thick, 500 µm long, and 100 µm wide) is 0.02 Nm^−1^, whereas for narrow fabricated silicon cantilevers (1 µm thick, 500 µm long, and 70 µm wide), the spring constant is 0.014 Nm^−1^ if we assume the linear scaling. For simplicity, the mechanical force analysis of the data for both sizes of the cantilever arrays was analyzed using *k* = 0.02 Nm^−1^. To examine the mechanical force generated using SAM-coated cantilevers under different pH conditions, the arrays were equilibrated in PBS solution at pH 4.8 until the signal became stable. Then, this was followed by injection of PBS solution at pH 9.0 under a constant flow rate of 30 µl min^−1^, leading to the deprotonation of the SAMs immobilized on the cantilever surface. The electrostatic repulsion between deprotonated SAMs resulted in stress changes, which caused cantilevers to bend. The deflection signal was directly converted into mechanical force by using the expression (4). For additional information, see the Supplementary Methods.

## Electronic supplementary material

Supplementary file

## Data Availability

All data generated or analyzed during this study are included in this published article (and its Supplementary Information file)
