# Supplementary material for: Modified cantilever arrays improve sensitivity and reproducibility of nanomechanical sensing in living cells
Source: Commun Biol. 2018 Oct 24;1:175. doi: 10.1038/s42003-018-0179-3 (PMC6200835; doi:10.1038/s42003-018-0179-3)
Supplement: Supplementary file 1 — Supplementary file [file 42003_2018_179_MOESM1_ESM.pdf]

Supplementary Methods

Two-dimensional (2D)-assembling of surface receptors

To test the hypothesis that mechanical forces arising from ligand-receptor interactions can be amplified to enable measurements of biologically relevant signals with high precision, we designed different patterns with a prescribed 2D-assembling of transduction arrays. The dip-pen lithography (DPN) and micro-contact printing (μCP) were used to create arrays parallel or transverse to the long axis of the conventional gold-coated nanomechanical cantilever arrays of (100 μm wide and 500 μm long IBM Rushlikon). The DPN has the advantage of enabling receptor patterns to be fabricated with nanometre precision[1-3] whilst μCP allows fabrication to be achieved more rapidly in a single step[4]. Parallel arrays were arranged as a 30 μm or 50 μm wide strip running centrally along the entire length of the cantilever covering 30%, 50% or 100% of the total surface area, respectively. Transverse arrays were arranged as 30 μm wide strips running along the width of the cantilever, and corresponding to 30% of the total surface area. A control cantilever had 100 μm wide strips running centrally along the entire of its length, and corresponding to 100% of the total surface area. SAMs of mercaptoundecanoic acid (MUA) and mercaptohexadecanoic acid (MHA) were used to create transduction arrays. MUA was used to create 30 μm wide strips for both parallel and transverse arrays as well as for control cantilevers. MHA was used to create 50 μm strips in the parallel arrays as well as for control nanomechanical cantilevers. These SAMs were chosen because of their capacity to be attached to a variety of receptor molecules, allowing the detection of a diversity of ligands. For example, in this study, MUA SAMs were attached to the VSR and anti-IgG antibody to enable detection of vancomycin (Van) and immunoglobulin G (IgG) respectively. Further, these SAMs are alkanethiols with less than 20 carbons and are

known to enable stable printing patterns with defined boundaries[4] and by functionalizing mechanical sensors with SAMs of carboxylic terminating groups, the scaffolds can be switched between 'oxidized' and 'reduced' states when the pH of the surrounding environment is changed[5]. To block nonspecific interactions, the un-patterned areas on the cantilever surface were passivated using SAMs of undecanethiol (UDT) in the case of MUA, and hexadecanethiol (HDT) for MHA.

Preparation of silicon substrates

Silicon substrates measuring 1 cm x 1 cm each was cleaned by incubating in a freshly prepared piranha solution, consisting of H_2SO_4 and H_2O_2 (1:1) for 20 min. They were then briefly rinsed in ultrapure water followed by a rinse in pure ethanol before drying on a hotplate at 75 °C. The silicon substrates were examined under an optical microscope to confirm their cleanliness before transferring to an electron beam evaporation chamber (BOC Edwards Auto 500, U.K.) where they were coated at a rate of 0.7 nms^{-1} with a 2 nm layer of titanium, which act as an adhesion layer, followed by a 20 nm layer of gold. Once the required thickness of gold was obtained, the silicon substrates were left in the chamber for 1-2 h to cool under vacuum.

Printing of transduction arrays

µCP stamps

Transduction arrays were printed on the gold-coated silicon substrates using SAMs of MUA as the printing ink. The protocol for printing MUA SAMs onto gold-coated silicon substrates is summarized in Supplementary Figure 1. The PDMS stamp was first cleaned by rinsing in pure ethanol before it was dried under nitrogen gas. The stamp was then impregnated with MUA by incubating in a freshly prepared solution

of MUA in ethanol at a total SAM concentration of 2 mM for 1 min. Excess MUA solution was removed from the PDMS stamp by blowing nitrogen gas over the PDMS stamp. The impregnated stamp was then placed in a conformal contact with the gold-coated surface for 2 min where gentle pressure (using a one penny coin) was applied on the PDMS stamp to allow close contact with a gold surface so that the MUA SAMs could diffuse from the PDMS stamp onto the substrate and enable uniform molecular printing.

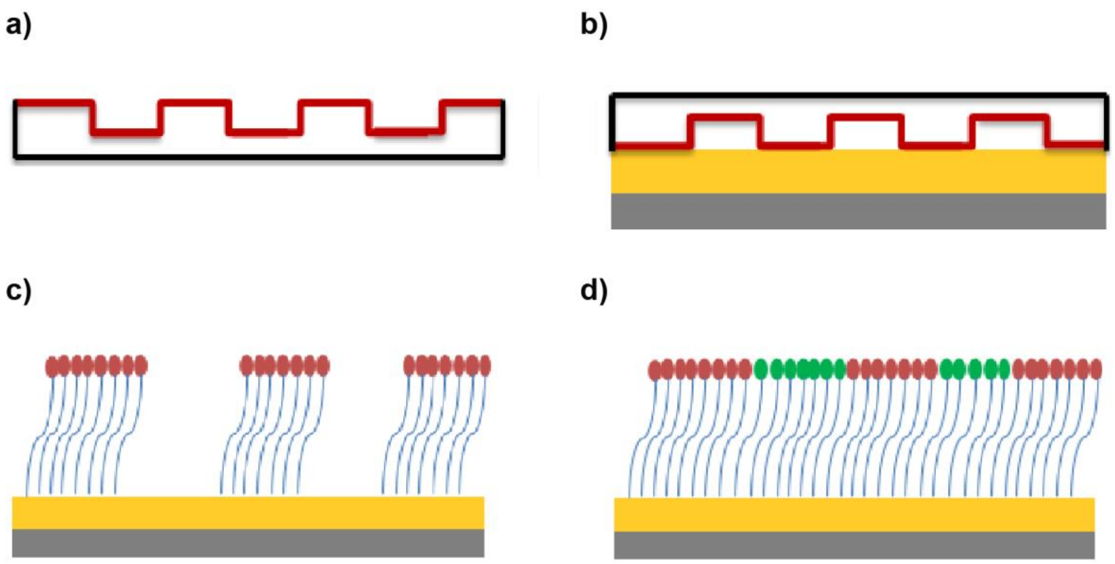

Supplementary Figure 1. Printing of transduction arrays using μCP stamps. **a** Schematic representation of a PDMS stamp in which it is inked with self-assembled monolayers (SAMs) of mercaptoundecanoic acid (MUA) (red). **b** The inked PDMS stamp is brought into conformal contact with the gold-coated (yellow orange) silicon substrate (grey) to enable the SAMs to diffuse from the PDMS stamp onto gold surface. **c** Schematic representation of MUA arrays arranged on a gold-coated silicon substrate generated by micro-contact printing. **d** The same MUA array following exposure to UDT SAMs in which the un-patterned areas on the gold-coated silicon substrate were passivated with UDT (green).

The PDMS stamp was removed after 2 minutes and the un-patterned areas on the gold-coated surfaces passivated by covering the entire surface with undecanethiol (UDT) for 20 min. This was followed by a rinse in pure ethanol and dried under nitrogen gas. Because of the fragile nature of cantilever arrays, the PDMS stamps were used to print SAMs onto the arrays only after mastering the procedure and if the printing was deemed satisfactory with the gold-coated silicon substrates.

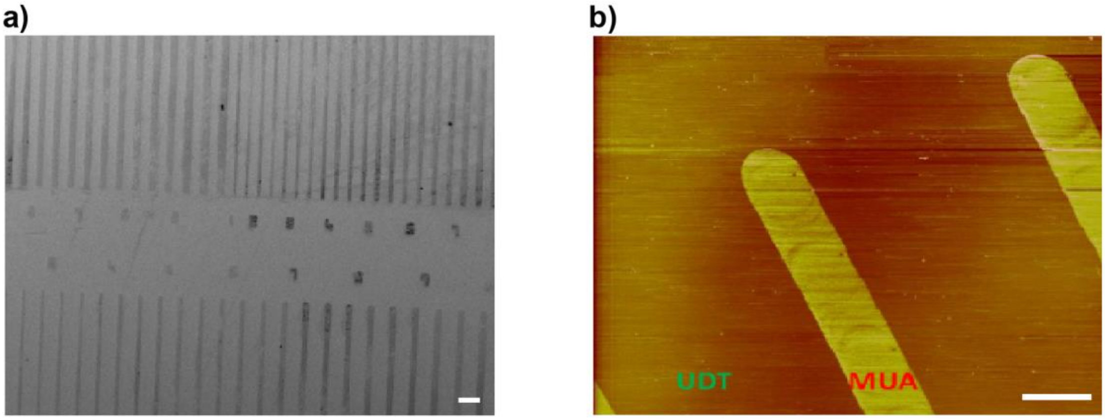

Supplementary Figure 2. Gold-coated silicon substrate printed using a μCP stamp. **a** Scanning electron microscopy (SEM) image showing patterns of self-assembled monolayers (SAMs) of mercaptoundecanoic acid (MUA) on gold-coated silicon substrate prepared by micro-contact printing (μCP). Scale bar, 100 μm. **b** Atomic Force Microscopy (AFM) image showing SAMs of MUA (yellow patterns) and undecanethiol (UDT) (dark orange un-patterned area) on gold-coated silicon substrate prepared by μCP. Scale bar, 10 μm.

The accuracy of the printed patterns on the gold-coated surfaces was checked by scanning electron microscopy. Supplementary Figure 2a shows a typical image of the PDMS stamps. The printed pattern was examined using atomic force microscopy (AFM) as shown in the Supplementary Figure 2b. In addition, the gold-coated

surfaces were exposed to moist air which preferentially condensed on the MUA coated surfaces. When viewed under a light microscope the hydrophilic MUA coated areas appeared dark in contrast to the hydrophobic UDT coated areas (Supplementary Figure 3a-c).

Dip-pen nanolithography

SAMs of MHA were printed onto gold-coated silicon substrates using dip-pen nanolithography. The printing process involved using a sharp scanning AFM cantilever tip to transfer SAMs of MHA as the printing ink directly onto the designated surface and to create the desired pattern. Supplementary Figure 3d shows the MHA pattern on a gold-coated silicon substrate which is clearly distinct from the underlying un-patterned areas.

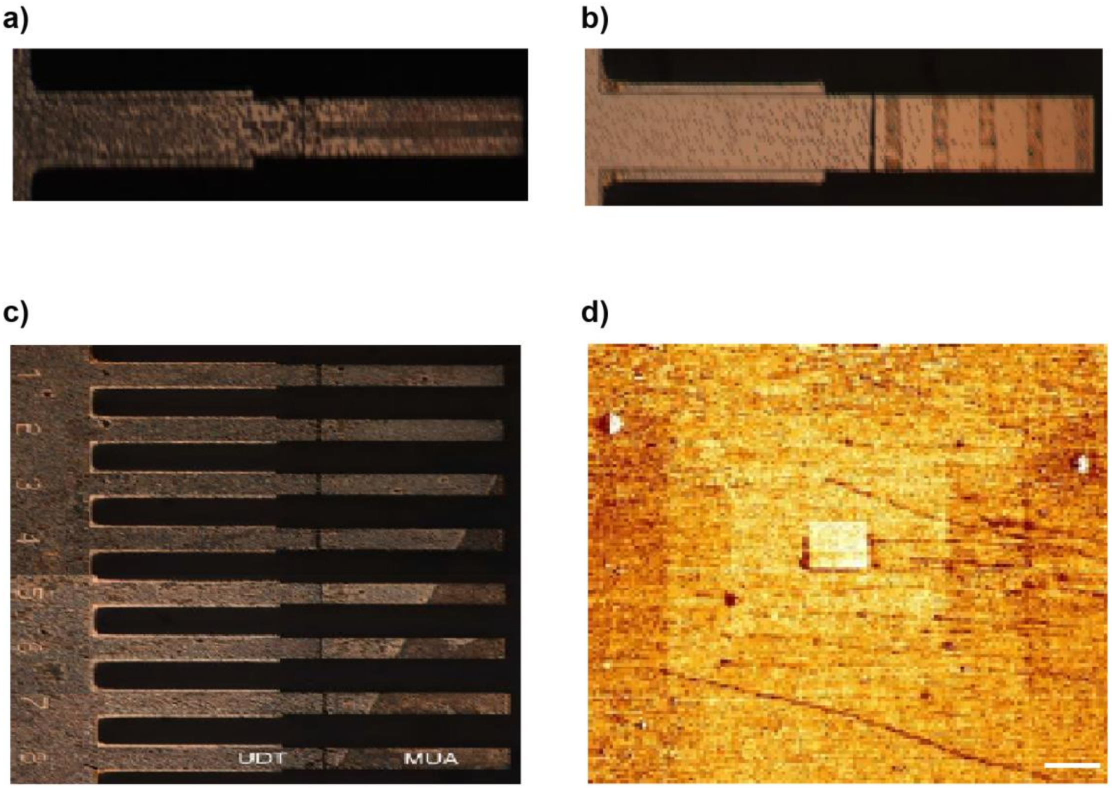

Supplementary Figure 3. Examination of the impact of mechanical connectivity on the signal response. **a** SAMs of MUA array networks arranged as a narrow strip (solid

line) running centrally along the entire length of the cantilever sensor and continuous with the hinge region. **b** MUA SAMs arranged in strips transverse to the long axis of the nanomechanical cantilever (solid lines) creating mechanical networks which are discontinuous with each other and with the hinge region. **c** Array networks of MUA SAMs (dark area) arranged continuously from the free end of the nanomechanical cantilever arrays but terminating at various distances from the hinge region. In **a-c** the un-patterned areas on the nanomechanical cantilever sensor (light background) were passivated using UDT SAMs to block nonspecific interactions. **d** Atomic Force Microscopy (AFM) image of MHA SAM patterns (central square) on the Au-coated silicon substrate prepared by dip-pen nanolithography (DPN). Scale bar, 1 μm.

Having established that the dip-pen nanolithography could be successfully used to write the array patterns on gold-coated silicon substrates, we then applied the same procedure to create transduction arrays on the nanomechanical cantilevers.

Location specific mechanical force in cell response

In Supplementary Figure 4, we show that when human epithelial cells (MDA-MB231) from malignant tumours and noncancerous cells (MCF10A) are injected into the cantilever liquid chamber, the cancer targeting peptides preferentially bind more to the breast cancer cells than to noncancerous epithelial cells. In addition, we find that the geometrical lengths of the regions covered by receptors and their location play a very significant role in the sensitivity of the mechanical force generated by cell-ligand binding interactions.

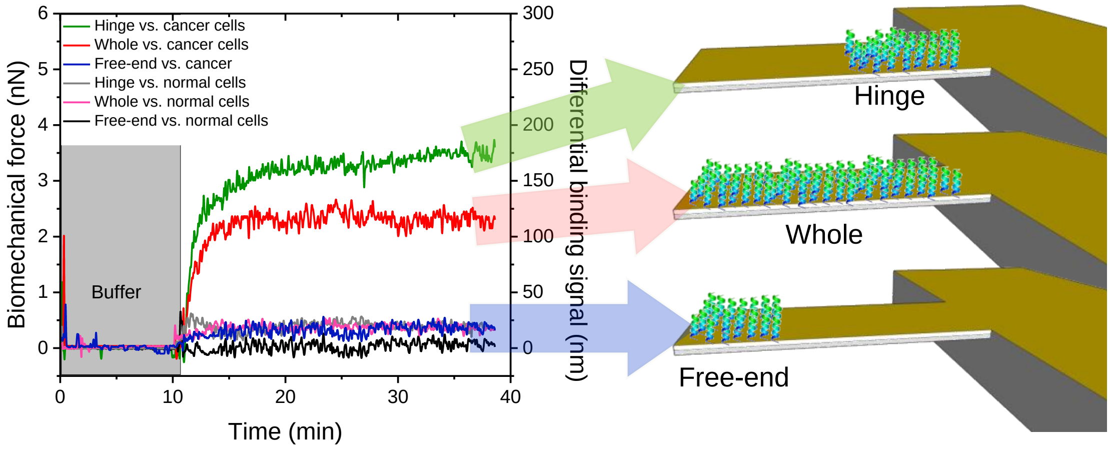

Supplementary Figure 4. Impact of receptor location on mechanical signaling. The detailed experiments depict the differential binding force of the receptor (decapeptide 18-4) to cancer cells while it is coated on the cantilever in three different geometrical locations. In the experiments, the cantilevers were exposed to breast cancer cells and/or normal cell lines at a concentration of 25 cells ml^{-1} in phosphate-buffered saline (PBS) solution. A relatively high mechanical force is observed when only the hinge region is coated with the peptide 18-4 compared to when the entire cantilever is coated with capture molecules. Similarly, negligible mechanical force is detected when the free-end of the nanomechanical cantilever is coated with capture molecules. The control experiment in which normal epithelial cells (MCF10A) were injected, exhibited insignificant mechanical force, indicating a weak binding strength of decapeptide capture molecules toMCF10A cells.

Derivation of expression for binding interactions

We first proposed that solvent effects are dominant factors important in pre-determining mechanical forces exerted by cells or molecules. Accordingly, we considered that an analyte can interact with a surface target and so the reactions are quantified by considering the distributions between analyte's concentrations in

solution [analyte] and membrane-bound receptors [R]. The reactions are quantified using

$$n(analyte) + R \underset{K_{off}}{\overset{K_{on}}{\rightleftarrows}} ((analyte)_n.R) \tag{1}$$

where n is the stoichiometric coefficient of the reaction and *analyte.R* is the bound complex. The expression between an analyte and receptor is

$$K_{\mathrm{d}}^{n}\left[((analyte)_n.R)\right] = [analyte]_{free}^{n}[R] \tag{2}$$

where K_{d} is the surface thermodynamic equilibrium dissociation constant. The total concentration of the surface receptor $[\mathrm{R}]_T$ is

$$[R]_T = [R]_{free} + [(analyte)_n.R] \tag{3}$$

Using equations (2) and (3), the fraction of an analyte bound at the surface is determined by the expression

$$\theta = \frac{[analyte]^n}{K_d^n + [analyte]^n} \tag{4}$$

where $\theta = [(analyte)_n.R]/[\mathrm{R}]_{\mathrm{T}}$ is the fraction of the surface occupied by the binding sites. If we assume that the change in the equilibrium mechanical force (ΔF_{eq}) involved in the analyte-receptor complex scales in direct proportion to the surface coverage ($\Delta F_{\mathrm{eq}} = F_{max}\theta$), then expression (4) is adjusted to yield

$$\Delta F_{\mathrm{eq}} = F_{\max}\left(\frac{[analyte]^n}{K_{\mathrm{d}}^n + [analyte]^n}\right) \tag{5}$$

Analysis mechanical force

The differential stress measurements obtained from cantilever chips are typically associated with multiple parameters including the number of repeated measurements, concentration and the number of cantilevers where each has eight individual cantilever arrays. The differential equilibrium mechanical forces for a wide range of concentrations of analytes (0.0005 – 1000 μM) or cells (10 – 1000 cells ml^{-1}) were determined using 4 chips for each ligand. For each analyte concentration, the arithmetic mean of the differential mechanical force data (ΔF_{eq}) across 4 chips was calculated using the equation

$$\Delta F_{eq} = \frac{\sum F_{diff}}{n} \qquad (6)$$

where n is the number of measurements, F_{diff} is the differential equilibrium mechanical force calculated by subtracting the *in-situ* reference force Fz_{ref} (MCH or PEG) coated cantilever from the absolute mechanical response Fz_{mea} obtained from VSR, anti-IgG, and cancer targeting peptides coated cantilevers. The standard deviation of the mechanical force data (σ) was calculated using the equation

$$\sigma = \sqrt{\frac{\left(\Delta F_{eq} - \Delta F_{diff}\right)}{n-1}} \qquad (7)$$

The standard error (SE) of the differential mechanical force for each analyte concentration was calculated using the equation

$$SE = \frac{\sigma}{\sqrt{n}} \qquad (8)$$

Supplementary references

1 Garcia, R., Knoll, A. W. & Riedo, E. Advanced scanning probe lithography. *Nat. Nanotech.* **9**, 577–587 (2014).

2 Lee, K. B., Park, S. J., Mirkin, C. A., Smith, J. C. & Mrksich, M. Protein nanoarrays generated by dip-pen nanolithography. *Science* **295**, 1702–1705 (2002).

3 Salaita, K., Wang, Y. & Mirkin, C. A. Applications of dip-pen nanolithography. *Nat. Nanotech.* **2**, 145–155 (2007).

4 Love, J. C., Estroff, L. A., Kriebel, J. K., Nuzzo, R. G. & Whitesides, G. M. Self-assembled monolayers of thiolates on metals as a Form of nanotechnology. *Chem. Rev.* **105**, 1103–1169 (2005).

5 Kaufman, E. D. et al. Probing protein adsorption onto mercaptoundecanoic acid stabilized gold nanoparticles and surfaces by quartz crystal microbalance and zeta-potential measurements. *Langmuir* **23**, 6053–6062 (2007).
